# Lip leishmaniasis: a case series with molecular identification and literature review

**DOI:** 10.1186/s12879-016-2178-7

**Published:** 2017-01-25

**Authors:** Iraj Mohammadpour, Mohammad Hossein Motazedian, Farhad Handjani, Gholam Reza Hatam

**Affiliations:** 1 Department of Medical Parasitology & Mycology, School of Medicine, Shiraz University of Medical Sciences, Shiraz, Iran; 2 Basic Sciences in Infectious Diseases Research Center, School of Medicine, Shiraz University of Medical Sciences, Shiraz, Iran; 3 Department of Dermatology, Molecular Dermatology Research Center, School of Medicine, Shiraz University of Medical Sciences, Shiraz, Iran

**Keywords:** Mucosal leishmaniasis, Case report, Lip, PCR, Phylogenetic analysis, Iran

## Abstract

**Background:**

Mucocutaneous leishmaniasis (MCL), a protozoan infectious disease, is very rare in Iran despite the endemicity of both cutaneous and visceral forms. It is transmitted by the *Phlebotomus* sand fly. The lip is considered one of the extraordinary sites. Lesions usually initiate with erythematous papules, slowly enlarges and then it ulcerates. The diagnosis of MCL encompasses epidemiological, clinical and laboratory aspects. Usually, the combination of some of these elements is necessary for the final diagnosis. So, lip leishmaniasis lesions can be challenging to diagnose.

**Case presentation:**

We presented seven rare cases of lip leishmaniasis. Tissue impression smear, culture, PCR and phylogenetic analysis were carried out for explicit diagnosis. Skin scraping investigation showed several *Leishmania* spp. amastigotes in the cytoplasm of macrophages. Culture examination was positive for *Leishmania* spp. PCR was positive for *L. major*, *L. tropica*, and *L. infantum*. Differential diagnosis includes orofacial granulomatosis, basal cell carcinoma, squamous cell carcinoma, and mesenchymal tumors. The cases were treated with systemic meglumine antimoniate (Glucantime^®^). No relapses were observed during 1 year of follow-up. Early detection of the infection are necessary in order to start effective treatment and prevent more serious complications.

**Conclusions:**

In this paper, we reported seven rare cases of lip leishmaniasis in Iran, emphasized the importance of clinical and diagnostic features of lesions, characterized the phylogenetic kinship of isolated parasites, and reviewed the literature on lip leishmaniasis.

## Background

Leishmaniasis is a group of infectious disease, caused by protozoan parasites belonging to the genus *Leishmania* (order Kinetoplastida). It is transmitted by sand flies of *Phlebotomus* and *Lutzomyia* species. Reservoirs are represented by a wide range of mammals, and more rarely, humans [1].

Leishmaniases present a wide spectrum of clinical manifestations including cutaneous (CL), diffuse cutaneous (DCL), mucocutaneous (MCL), and visceral leishmaniasis (VL) [1]. CL is endemic in half of the 31 provinces of Iran, and is a great health problem [2].

From the clinical vista, CL is characterized by skin lesions that vary in presentation from papules to plaques to ulcers. These lesions can cause varying amounts of scarring depending on the number and size of lesions [3]. Although localized lymphadenopathy may be present in the area of the cutaneous lesion, systemic complications from this form of leishmaniasis are rare [3].

MCL is characterized by the scatter of skin ulcers to encompassing tissues, specifically inner nostril wall to larynx and mouth [3]. Moreover, there may be cases in which the MCL would be seen without cutaneous lesions. In fact, in 30% of MCL cases, patients do not recall the presence of a primary cutaneous lesion [3]. Primary mucosal leishmaniasis is very rare in Iran in spite of high prevalence of cutaneous and visceral forms. The lip involvement is very rare and may imitate orofacial granulomatosis such as Crohn’s disease, sarcoidosis, foreign body giant cell granuloma and Melkersson-Rosenthal syndrome [4]. Other differential diagnosis includes basal cell carcinoma, squamous cell carcinoma, mesenchymal tumors, and mycotic infections. Because of its heterogeneous and underestimated clinical presentation [5, 6], MCL is often perplexed and remains a diagnostic challenge for the clinicians and scientists.

The diagnosis of MCL includes epidemiological, clinical and laboratory aspects. Generally, the combination of some of these constituents is necessary for the final diagnosis [7]. Unfortunately, delayed diagnosis and the lack of consensus on optimal treatment can frequently lead to inappropriate management of the disease [8].

In this report, we presented seven rare cases of lip leishmaniasis in Iran, emphasized the importance of clinical and diagnostic features of lesions, characterized the phylogenetic relationship of isolated parasites, and reviewed the literature on oral leishmaniasis. Also, we highlighted the important role of the clinicians in the diagnosis of perioral leishmaniasis lesions, which are unusual and can be confounded with other diseases, especially in individuals living or traveling in certain geographic regions where the parasite is endemic.

## Case presentation

### Case 1

**Fig. 1:**
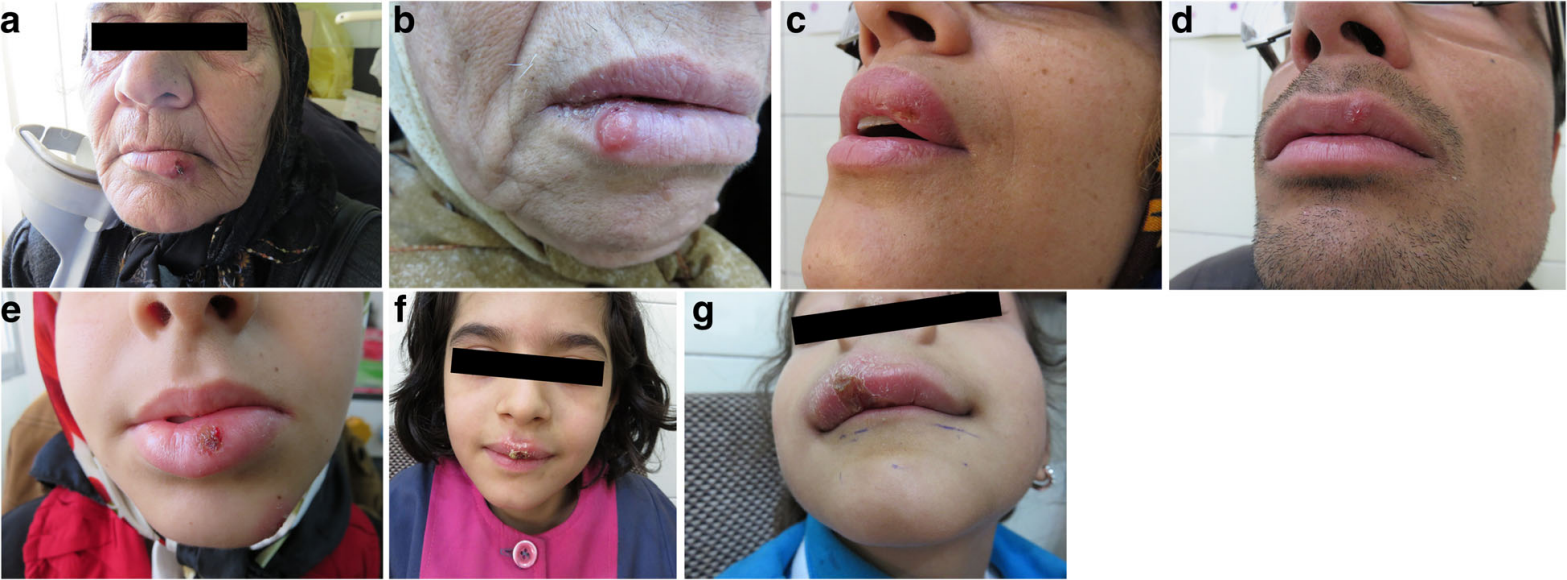
**a** An erythematous, calloused, crusted left lower lip ulcerative plaque in patient 1. **b** A round shiny and *red* colored nodular lesion with hard consistency in the right inferior lip of patient 2. **c** An erythematous, edematous, and elliptical ulcerative plaque of the left upper lip with overlying *yellow* crusting lesion extending to the wet line in patient 3. **d** Sore swelling, with well-defined edges and scaling, involving the left side of the upper lip in patient 4. **e** A deep *red-colored* ulcer covered with mild hemorrhagic crusts and severe swelling of the lower lip in patient 5. **f** Mild swelling of the superior lip, in addition to ulceration, *yellow crusting*, scaling and bleeding in patient 6. **g** A severe swelling cheilitis, with crusting, scaling and fissuring lesion of the right superior lip in patient 7

A 65-year-old woman was referred to the Fajr Health Center. The chief complaint was a lip lesion that began 2 months prior as a slowly enlarging nodule. Patient reported that the lesion began as a small soft mass, which ulcerated later. Clinical examination showed a necrotic ulcer of the left lower lip vermillion. The lip was erythematous and edematous from midline to the left commissure mediolaterally (Fig. 1a). With suspicion of basal cell carcinoma and squamous cell carcinoma, skin scraping, culture, and PCR were accomplished. Touch impression smears and culture were positive and the parasite was characterized by PCR as *L. tropica*.

### Case 2

A 75-year-old woman came to the Dermatology Clinic of Saadi Hospital complaining about a lesion on his right lower lip. She reported that the lesion appeared about 3 months before. The clinical examination revealed a round nodular lesion in the right lower lip which was slightly shiny and red colored. Palpation of the lesion revealed a firm consistency (Fig. 1b). The differential diagnosis of a nodular lesion of the lip included: neoplasia (basal cell carcinoma), and tumors from mesenchymal origin (fibroma, lipoma, and neuroma). Giemsa stain and culture were negative. PCR depicted the parasite as *L. tropica*.

### Case 3

**Fig. 2:**
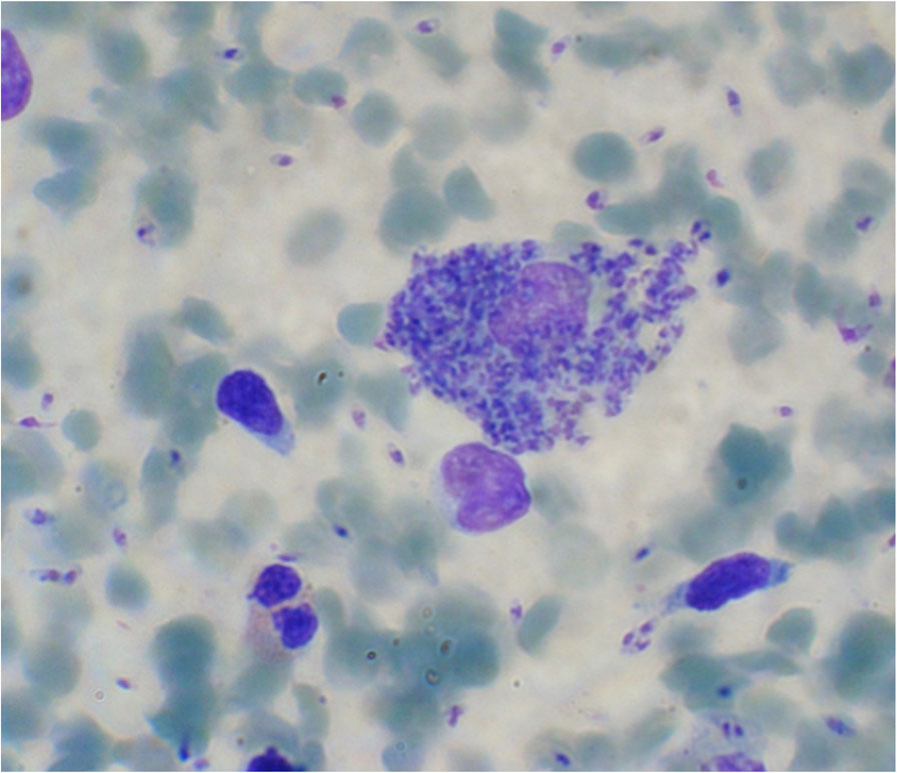
Touch impression smear in patient 3. Numerous intracellular and scattered extracellular amastigotes are present (Giemsa stain; original magnification, ×1000). Kinetoplasts are visible in many amastigotes

A 40-year-old, generally healthy woman complained of persistent lip enlargement and sore that appeared after several days of fever and had been present for 2 months. At clinical examination, the left side of the upper lip appeared erythematous and distended, with overlying yellow crusting lesion extending to the wet line. No lymphadenopathy was associated (Fig. 1c). Giemsa-stained smears and culture were positive (Fig. 2). PCR identified the parasite as *L. major*.

### Case 4

A 28-year-old man expressed displeasure about a swelling localized to the left side of the upper lip. The patient also stated that the swelling had appeared about 2 months before and that it rapidly enlarged up to the current morphology and size. Clinical examination showed the presence of a swelling which involved the left side of the upper lip. The surface of the swelling was slightly erythematous and irregular, because of the presence of lesion and scaling (Fig. 1d). Consistency was parenchymatous-hard. With suspicion of mycotic infection, laboratory tests were carried out. Slit dermal smears and culture were positive. PCR characterized the parasite as *L. tropica*.

### Case 5

A 10-year-old girl was referred to the Dermatology Clinic of Saadi Hospital. The patient had become infected with CL on the left side of her mentum 3 months before. Three weeks prior to referral, the lesion had spread to the mucosal part of the lower lip. The patient improved diffuse severe swelling of the lower lip. On physical examination, erythema, ulceration, and scaling of the left side of the mentum were detected. There was a diffuse swelling cheilitis of the lower lip extra to ulceration and bleeding of the labial mucosa (Fig. 1e). Giemsa-stained smears and culture were positive. PCR identified the parasite as *L. major*.

### Case 6

An 8-year-old girl presented to the Leishmaniasis research laboratory of the Fajr Health Center with complaint of wound on her upper lip. The lesion started 3 months prior as a small nodule in the center of the upper lip which slowly enlarged over a few weeks and then ulcerated. Clinical examination showed an indurated, crusted, and scaled necrotic ulcer with swelling on the midline of the upper lip, extending to the vermilion. There was purulent discharge from the lesion (Fig. 1 f). With suspicion of cheilitis granulomatosa, slit dermal scraping, culture and PCR were performed. Giemsa-stained smear and culture were positive. PCR portrayed the parasite as *L. major*.

### Case 7

A 4-year-old girl presented with a 2 month history of increasing swelling and painful ulcer on the right upper lip. On clinical examination, an erythematous infiltrated, crusted, scaled and fissured plaque of the upper lip leading to macrocheilitis was observed. Cervical lymph nodes were noticed to be enlarged (Fig. 1g). With suspicion of granulomatous macrocheilitis; slit impression smear, culture and PCR were achieved. Just PCR was positive and characterized the parasite as *L. infantum*.

The studied patients were examined in the Dermatology Clinic of Saadi Hospital and Fajr Health Center from January 2014 to the end of December 2015. All patients were came from endemic regions of Fars province. A structured questionnaire was used to collect clinical and therapeutic data from all patients.

The margin dermal scrapings were prepared with a no. 15 disposable sterile surgical blade (Unicut, Chicago, USA) to make a slit in the border of the lesion. Dermal tissues were stained with Giemsa (Merck, Darmstadt, Germany), and exhibited amastigotes by light microscope. Employing Giemsa, amastigotes are seen within the cytoplasm of macrophages as pale blue oval bodies with a dark blue nucleus and a small rod-shaped kinetoplast with a specified mitochondrial frame that contains extra-nuclear DNA.

In addition, an aspirate from the active indurated margins of the same lesions was transferred to two tubes of the modified NNN culture medium. Modified NNN medium consists of two phases, horse blood agar base and an overlay Locke’s solution. The specimens were inoculated into the liquid phase of the biphasic medium and incubated. Positive cultures were run in RPMI-1640 medium (Gibco, Frankfurt, Germany) supplemented with 10% heat inactivated FCS (Gibco, Frankfurt, Germany), 100 U/mL penicillin, and 100 μg/mL streptomycin (Gibco, Frankfurt, Germany) for mass cultivation. Promastigotes were harvested and kept at -20 °C until used.

Total genomic DNA was extracted from each clinical sample using the AccuPrep^®^ Genomic DNA Extraction Kit (Bioneer, Daejeon, Korea), according to manufacturer’s instructions. The quantification and quality control of the DNA extraction procedures were performed using a nano spectrophotometer (NanoDrop 1000, Thermo Fisher Scientific, Waltham, Massachusetts, USA). The DNA was stored at −20 °C until being used.

**Fig. 3:**
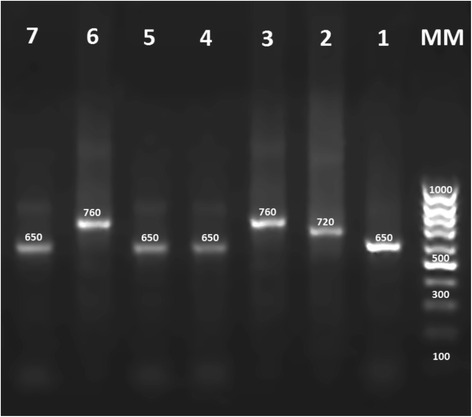
Electrophoresis of PCR products of DNA extracted from positive smears and cultures. The seven lanes contained the products from positive controls of *L. tropica* (lane 6) and *L. major* (lane 7), lip lesions due to *L. major* (lanes 1, 4, 5), *L. infantum* (lane 2), *L. tropica* (lane 3), and a molecular size marker (MM)

The conserved area of the minicircle kDNA from the *Leishmania* species was amplified by Semi-Nested PCR. The primers LINR4 (forward) (5’-GGG GTT GGT GTA AAA TAG GG-3’), LIN17 (reverse) (5’-TTT GAA CGG GAT TTC TG-3’), and LIN19 (reverse) (5’-CAG AAC GCC CCT ACC CG-3’) were used for amplification [9]. Standard PCR was carried out with 40 cycles, each consisting of 30 s at 94 °C, 30 s at 52 °C (for LINR4 and LIN17) or 58 °C (for LINR4 and LIN19), 1 min at 72 °C, and a final extension at 72 °C for 10 min in an Eppendorf thermal cycler (Hamburg, Germany). PCR products were visualized by UV after electrophoresis on 1.5% agarose gel using TAE buffer and staining with GelRed (Biotium Inc., Hayward, CA). All primers were synthesized by Macrogen Genomics Laboratories (Macrogen, Seoul, Korea). A 650-bp fragment was amplified for *L. major*, whereas a 760-bp and 720-bp fragments were amplified for *L. tropica* and *L. infantum*, respectively (Fig. 3).

**Fig. 4:**
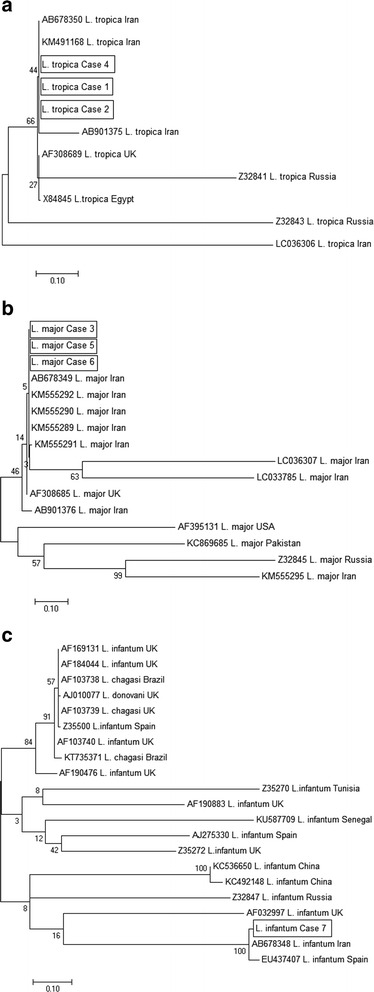
**a**, **b**, **c** Phylogenetic relationship among various *Leishmania* species to each other as inferred by Maximum Likelihood tree based on minicircle kDNA gene. Numbers on branches are percentage bootstrap values of 1,000 replicates. All positions with less than 95% site coverage were eliminated. The evolutionary distances between sequences were computed using the Maximum Composite Likelihood method and are in the units of the number of base substitutions per site. The scale bar indicates an evolutionary distance of 0.10 nucleotides per position in the sequence. The reference sequences accession numbers are inserted. Evolutionary analyses were conducted in MEGA-7

The PCR products were purified by using QIAquick Gel Extraction Kit (QIAGEN, Hilden, Germany) and sequenced through the sequencing service of Macrogen Genomics Laboratories (Seoul, Korea). The resulting sequences were aligned and compared with those of existing sequences related to *Leishmania* in GenBank. A Maximum Likelihood (ML) tree was constructed using the MEGA-7 program [10], and genetic distances were calculated with the Maximum Composite Likelihood method. The sequences of three patients of *L. tropica* showed 100% identity to the published isolate IranJWtrop (AB678350). Also, these three isolates showed 99% identity to the published isolates from UK, Egypt, and Iran (AF308689, X84845, and KM491168) (Fig. 4a). The sequences of three patients of *L. major* showed 100% identity to the published isolate IranJWmaj (AB678349), In addition, the three *L. major* showed 99%, 99% and 98% identity to the published isolates from UK and Iran (AF308685, KM555292, and KM555295) respectively (Fig. 4b). The sequence of *L. infantum* showed 99% identity to the published isolate IranJWinf (AB678348). Plus, this isolate showed 98% identity to the published isolate from Spain (EU437407) (Fig. 4c).

The patients received intramuscular injections of Meglumine antimoniate (Glucantime^®^; Sanofi-Aventis, Paris, France), 20 mg/kg/day over a 3-week period with good results [7, 8]. All lesions resolved after 3 weeks of therapy. After 1 year of follow up, the patients were completely normal and no relapses were observed.

## Discussion

Cutaneous leishmaniasis is a protozoan-induced disease known to affect people in 98 tropical, subtropical, and Mediterranean countries [1]. The incubation period of CL ranges from 2 weeks up to several months, and a wide spectrum of clinical presentations arranging from cutaneous ulceration to various degrees of mucosal involvement [3]. CL is the most prevalent and is characterized by the presence of ulcers with a well-defined erythematous border and a central crust that is often hemorrhagic and located in exposed areas of the body [3, 7].

In recent years, there has been an intensifying in the number of reports for rare variants of CL in Old World [11]. MCL most usually influences the upper respiratory tract with lesions principally in the oral and nasal mucosa and occasionally in the laryngeal and pharyngeal mucosa [12–18]. These lesions are ordinarily associated with nasal congestion, pain, erythema, edema, halitosis, bleeding, serous rhinorrhea, epistaxis, dysphagia, and dysphonia [19]. The importance of the MCL is in the possibility of having presentations that may determine destructive, disfiguring and disabling injuries, with major repercussions in psychosocial aspect of the individual [19].

The first commitment and sole mucosal involvement of the lip region is very uncommon. Lip leishmaniasis is characterized clinically by the gradual and proceeding expansion of one or both lips and macrocheilitis is the final presentation [20–23]. A papule, nodule or plaque often demonstrate within the swelling undergoes an ulceration which may be covered by crusts and scaling [20–23]. The consistency of the entire lesion is parenchymatous-hard [22, 23]. Lip involvement in leishmaniasis may result from direct extension of nearby skin lesions, as occurred in case 5, or from hematogenous or lymphatic dissemination of *Leishmania* amastigotes from the skin [19]. Patients with lip leishmaniasis are in good general health, however, case 7 in our study was noticed to have cervical lymphadenopathy. This is in accordance with a previous report of localized leishmaniasis of the larynx [24]. It has been hypothesized that *L. infantum* strains involved in isolated ML, have developed different resistance to high/low temperature, attaining the capability to live electively in mucous membranes [6]. In case 5, nodular crusted plaque localized on the left side of the mentum was representative for CL and it was the clue for us that lip lesion could also be a *Leishmania* lesion.

In MCL, the clinical demonstration at the perioral site is atypical and can be deceiving. Accordingly, clinical diagnosis of lip leishmaniasis is frequently a challenge with a significantly delayed diagnosis or even an erroneous clinical diagnosis of malignancy [12, 19, 25]. The most important diseases that must be taken into account in differential diagnosis are herpes labialis [26], syphilitic chancre [27], Melkersson-Rosenthal syndrome [28], orofacial granulomatosis [28], cheilitis granulomatosa [29, 30], Wegener granulomatosis [12, 19, 25], oral Crohn’s disease [31], sarcoidosis [32, 33], skin tuberculosis [34], discoid lupus erythematosus [34], lymphoma [34], foreign body giant cell granuloma [12, 19, 25, 28], leprosy [35], mycotic infection [36], fibroma, lipoma, and neuroma [37], basal cell carcinoma [38], and squamous cell carcinoma [34, 39]. The diagnosis is easier to make if typical CL lesions are present elsewhere on the skin. However, this was observed in only case 5.

Identification of *leishmania* parasites in dermal macrophages by skin biopsy or dermal scraping can confirm the diagnosis. When performing the slit smear, it is important to scrape tissue from the inside of the lesion and from the edges of the cut. However, in chronic lesions, parasites may be scarce. Therefore, failure to visualize amastigotes on histopathology does not exclude the diagnosis of MCL. In this context, culture, IFA and PCR can be amongst significant diagnostic techniques [7].

Detection of the kDNA of *Leishmania* spp. by semi-nested PCR in the oral mucosal specimen and clinical course of the disease verified the diagnosis of MCL. PCR is now the diagnostic method of choice for molecular confirmation of cases of MCL [40]. In this study, *L. tropica*, *L. major*, and *L. infantum* were the identified species in this case series.

Involvement of the lip can be treated with intralesional injections, systemic treatment, or their combination depending on the clinical manifestation and infective species. Intralesional injections are associated with burning sensation, pain, inflammation, and vasovagal reaction [41]. Systemic treatment is usually more effective than local, although less tolerated because of increased toxicity. The widely used therapeutics for treatment of mucosal leishmaniasis are: pentavalent antimony compounds (Pentostam^®^ and Glucantime^®^), liposomal amphotericin B (Ambisome^®^), pentamidine, oral azole compounds, and miltefosine [7, 8, 41]. Recommended follow-up times vary, although 12 months has been suggested for CL and MCL [12].

## Conclusion

In conclusion, we highlight a case series of lip leishmaniasis which are rare and can be confused with other diseases. In addition, the present report emphasizes the importance of a multidisciplinary approach in the diagnosis and treatment of lip leishmaniasis. These unusual clinical presentations of leishmaniasis should be considered in differential diagnosis of rebellious lip lesions by physicians practiced both in endemic and non-endemic regions, since MCL is ascending in these areas due to increasing rate of traveling, as tourists or military personnel, to endemic regions. Clinician should kept in mind leishmaniasis in the differential diagnosis of orofacial granulomatosis and other inflammatory and neoplastic diseases.

## Abbreviations

CL: Cutaneous leishmaniasis
DCL: Diffuse cutaneous leishmaniasis
MCL: Mucocutaneous leishmaniasis
VL: Visceral leishmaniasis
